# Immune Landscape of Colorectal Cancer Tumor Microenvironment from Different Primary Tumor Location

**DOI:** 10.3389/fimmu.2018.01578

**Published:** 2018-07-10

**Authors:** Longhui Zhang, Yuetao Zhao, Ying Dai, Jia-Nan Cheng, Zhihua Gong, Yi Feng, Chengdu Sun, Qingzhu Jia, Bo Zhu

**Affiliations:** ^1^Institute of Cancer, Xinqiao Hospital, Army Medical University, Chongqing, China; ^2^Chongqing Key Laboratory of Tumor Immunotherapy, Chongqing, China

**Keywords:** right-side colon cancer, left-side colorectal cancer, tumor microenvironment, immunophenotype, therapeutic implications

## Abstract

To define differences in tumor microenvironment (TME) immune phenotypes between right and left colorectal cancers (CRCs) and explore their therapeutic implications. Gene expression profiling and clinical characteristics of patients with CRC were retrieved from The Cancer Genome Atlas data portal. Immune cell infiltration was estimated based on single-sample gene set enrichment analysis. CRCs tissue microarrays (TMAs) containing 90 consecutive cases of surgical samples were used for validation. Expression of CD8A and VEGFA was confirmed by immunohistochemistry (IHC) analysis with TMAs, and overall survival (OS) was analyzed. Expression profiling data demonstrated that CRC immune microenvironment from right side tumor was characterized as increased infiltration of immune cells with enhanced cytotoxic function, based on higher cytotoxic activity scores (CYT) and interferon-γ signatures. Expression of VEGFA, which could be neutralized by bevacizumab, was associated with decreased levels of activated CD8^+^ T-cells, Th1 cells, and PRF1 expression on the right side, but not on the left side. IHC analysis of TMAs further confirmed an inverse correlation between CD8A and VEGFA expression, and revealed a favorable OS for patients with CD8A^Hi^VEGFA^Lo^ disease among right-side CRCs. For the left side, higher CD56^bright^ natural killer cell infiltration and active 4-1BB/IFN-ɑ signaling, which could providing a favorable condition for cetuximab-mediated antibody-dependent cell-mediated cytotoxicity effect, was present in a cohort with extended OS. In the TME, features of immune phenotype sidedness were identified, providing an implication for differential responses to bevacizumab/cetuximab treatment. In addition, a new avenue for innovative experimental design and combinational immunotherapy to treat CRC patients was suggested.

## Introduction

Colorectal cancer (CRC) is one of the leading causes of cancer mortality worldwide (1). Growing evidence suggests that CRC should be considered a heterogeneous disease; specifically, proximal (cecum to transverse colon) and distal (splenic flexure to rectum) CRC show various biological and clinical differences including embryonic origin, vascular supply, and main physiologic function (2), which affects disease prognosis (3). Moreover, a series of clinic trials and pooled analysis (4–7) have shown that the location of the primary tumor can be both predictive and prognostic regarding the response to anti-EGFR and anti-angiogenic agents in metastatic colorectal cancer (mCRC). The strongest evidence for the predictive value of primary tumor sidedness and response to EGFR inhibitors is in the first-line treatment of patients in the phase III CALGB/SWOG 80405 trial. The study showed that patients with all RAS wild-type, right-sided primary tumors had longer overall survival (OS) if treated with bevacizumab than if treated with cetuximab in first line, whereas patients with all RAS wild-type, left-sided primary tumors had longer OS if treated with cetuximab than if treated with bevacizumab (5). Specifically, worse prognosis is associated with right-side tumors, when compared with that with left-side tumors, in patients with RAS wild-type mCRC (5, 6). Accordingly, for right-side mCRC, chemotherapy plus bevacizumab seems to be a treatment option. However, the underlying mechanism through which CRC location differentially affects the response to anti-EGFR or anti-angiogenic therapies remains unknown.

Increasing evidence suggests that tumor progression and recurrence are not only governed by the genetic changes inherent to cancer cells but also by tumor microenvironment (TME) factors (8–10). Numerous studies have confirmed that immune cells in the TME modulate cancer progression and are attractive therapeutic targets (11–13). Moreover, the effects of infiltrating immune cells on prognosis have been extensively reported (14–16). Accordingly, we propose that right- and left-side CRC have different immune landscapes, and further, different immune landscape might lead to different prognoses and treatment responses. In this study, we comprehensively compared differences in the immune landscapes between right- and left-side CRCs. To some extent, our findings explain the discrepancies in the prognoses and responses to anti-VEGF and anti-EGFR agents that occur with different primary CRC locations (5, 17, 18). Moreover, the present findings suggest a novel combinational immunotherapy strategy for CRCs of different locations.

## Materials and Methods

### Data Sources

The RSEM-normalized RNA-Seq or microarray datasets for the The Cancer Genome Atlas (TCGA) colon adenocarcinoma (COAD, *n* = 467) cohort and rectum adenocarcinoma (READ, *n* = 172) cohort were downloaded. In total, 638 CRC patients, for whom transcriptome profiling data and clinical characteristics were available, were enrolled in this study. In addition, TCGA data sets and Molecular Signature Database (MSigDB) gene sets were downloaded from https://genome-cancer.ucsc.edu and the Gene Set Enrichment Analysis browser, respectively. The study involved mutation annotation files (MutSig 2.0) that were acquired from the Broad Institute TCGA GDAC Firehose’s analysis-2015-04-02.^1^

### Gene Signatures

We adopted a previously described procedure to determine the infiltration of immune cells in COAD/READ (19). We obtained the marker gene set for immune cell types from Bindea et al. (20). To calculate single-sample gene set enrichment, we used the GSEA program to derive the absolute enrichment scores of previously experimentally validated gene signatures.

### Implementation of Single-Sample Gene Set Enrichment Analysis (ssGSEA)

In brief, the infiltration levels of immune cell types were quantified by ssGSEA in R package gsva. The ssGSEA applies gene signatures expressed by immune cell populations (20) to individual cancer samples (21). The deconvolution approach used in our study included 27 immune cells that are involved in innate immunity [natural killer (NK) cells, CD56^dim^ NK cells, CD56^bright^ NK cells, plasmacytoid dendritic cells (DCs), immature DCs, activated DCs, neutrophils, monocytes, mast cells, eosinophils, and macrophages] and in adaptive immunity (immature B cells, activated B cells, central memory CD4^+^ T, effector memory CD4^+^ T-, activated CD4^+^ T, central memory CD8^+^ T, effector memory CD8^+^ T-, activated CD8^+^ T-, NK T-, T follicular helper, Tγδ, Th1, Th2, Th17, and Treg). The observation of T-cell infiltration score (TIS) was defined as the average of the standardized values for CD8^+^ T, central memory CD4^+^ T, effector memory CD4^+^ T-, central memory CD8^+^ T, effector memory CD8^+^ T-, Th1, Th2, Th17, and Treg cells. The obtained CYT score rule from the data set of Rooney et al. (22) consisted of cytolytic genes (calculated as geometrical mean of PRF1 and GZMA). The CD8^+^ T/Treg ratio was the digital ratio of ssGSEA scores for these two cell types. Signaling pathway was evaluated based on ssGSEA (23) according to previously report (24). Gene set for “41BB signaling pathway” and “Interferon-a response” was retrieved from MSigDB (25).

### Clinical Information

For COAD and READ, we downloaded clinical files from Genomic Data Commons Data Portal.^2^ The sample sizes of different types of analysis are different depending on required data availability.

### Survival Analysis

Survival outcome analysis modeled results in reference to the patient OS; specifically, events were defined as death by any cause, and time was accurate to the day. *p*-Values were obtained from univariate Cox proportional-hazards regression models using the R package survival. All Kaplan–Meier survival curves were drawn using the survfit function in the survival package and were plotted together with the *x*-axis representing time, and ranging from 0 to 4,000 days.

### CRC Tissue Microarray (TMA)

The CRC TMAs contained 90 consecutive cases of surgically resected stages I, II, and III CRC. For each case, one core, 1.5 mm in diameter, was taken at random from the tumor, and an additional 1.5-mm diameter core was taken from histologically normal colonic mucosa. Whole TMAs were stained using the standard protocol. In brief, antigen retrieval was performed using EDTA, samples were incubated with the following primary antibodies: recombinant mouse monoclonal anti-human CD8 alpha (1:400 dilution, Abcam; ab199016) and recombinant rabbit monoclonal anti-human VEGFA (1:400 dilution, Abcam; ab52917) for 14 h at 4°C. A negative control was performed by omitting the primary antibody.

### Measurement of CD8 Alpha Cell Number and VEGFA Density/Area

After staining for CD8 alpha and VEGFA, slides were scanned with a high-resolution scanner (ScanScope XT; Aperio) at 20× magnification. Image analysis software (Image-Pro Plus, version 6.0.0.260) was used to evaluate the number of CD8 alpha cells and density/area of VEGFA. CD8^+^ T-cells and VEGFA density/area were assessed in three locations for each microarray as follows: the intratumoral compartment (within the tumor cell nests) and within the adjacent stroma (defined as CD8 cells within one tumor cell diameter of the tumor). The total number of CD8^+^ T-cells and the density/area of VEGFA were determined by combining the counts for the two compartments. Scores were randomly re-examined by the same investigator after a period of time to ensure reproducibility.

### Statistical Methods

A two-sided Mann–Whitney was performed with the R functions *wilcox.test* for nonparametric categorical comparisons. Because we know that the ssGSEA scores are approximately normally distributed, two-tailed *t*-tests and one-way ANOVAs were performed for continuous comparisons. The CD8^+^ T/Treg ratio represents the digital ratio of ssGSEA scores for these two cell types. Unsupervised clustering for tumor samples, immune cell types, and genes was performed by hierarchical clustering with Pearson’s correlation coefficients.

## Results

### Immune Phenotype Landscape in TME From CRCs

**Figure 1:**
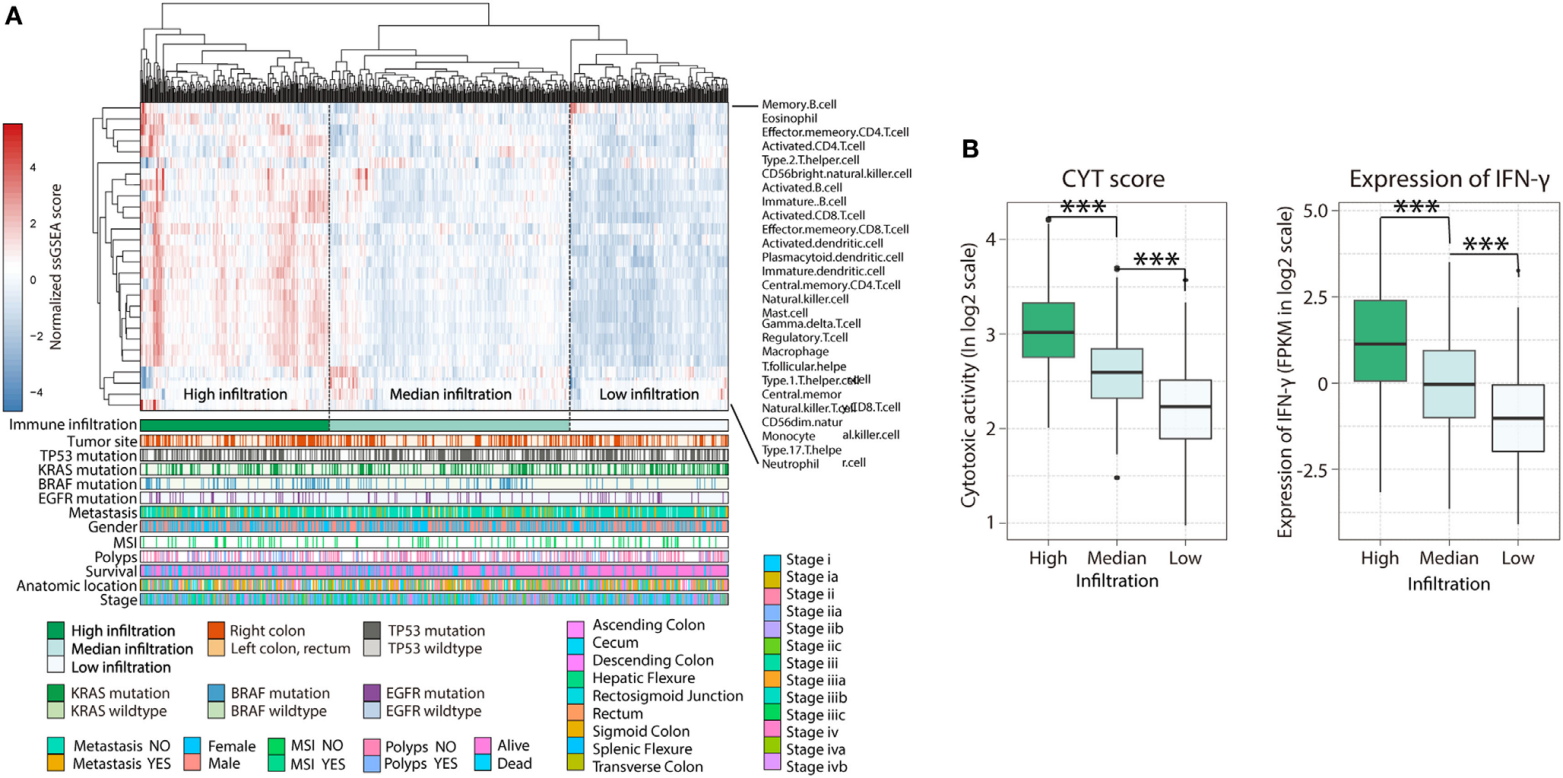
Immune landscape of colorectal cancer. **(A)** Unsupervised clustering of 638 patients from the The Cancer Genome Atlas cohort using single-sample gene set enrichment analysis scores from 27 immune cell types. Primary tumor location, mutation status of *EGFR, BRAF, KRAS*, and *TP53*, metastasis, gender, MSI, polyps, survival, anatomic location, as well as stage were annotated in the lower panel. Hierarchical clustering was performed with Euclidean distance and Ward linkage. Three distinct immune infiltration clusters, here termed high infiltration, median infiltration, and low infiltration, were defined. **(B)** Relative cytolytic score (CYT) and expression of IFN-γ between immune infiltration high, median, and low tumors clustered by overall immune cell infiltration. Two-tailed Student’s *t*-tests were used for all analyses. Error bars represent the mean ± SEM. *p < 0.05; **p < 0.01; ***p < 0.001; *p* ≥ 0.05, not significant.

Diverse immune cell populations infiltrate the TME and modulate the antitumor response (*via* activation or suppression). To compare immunophenotypes between CRCs from the left and right side, we first assessed the spectrum of immune cell infiltration. The ssGSEA (21) approach was utilized to deconvolve the relative abundance of each cell type based on expression profiling data retrieved from the TCGA database. For this, 638 CRC patients, for whom transcriptome profiling data and clinical characteristics were available, were included in this study. Based on this analysis, we found significant heterogeneity in terms of infiltration of numerous immune cell types among the cohort (Figure 1A); this was in accordance with a recent publication reporting tumor-infiltrating lymphocyte subpopulations in CRC (26). To facilitate further characterization, unsupervised clustering was applied to categorize the cohort into three infiltration subgroups—termed high (H, *n* = 214), median (M, *n* = 261), and low (L, *n* = 163) infiltration. Considering the concomitant infiltration of activating and suppressive immune cell types, we investigated whether higher immune cell infiltration correlated with elevated levels of cytotoxic function. To this end, cytolytic activity (CYT), which can serve as a surrogate index for quantifying the magnitude of antitumor response (22), and the expression of IFN-γ, an effector molecule released by activated T-cells, was examined. Patients with high infiltration status showed the highest IFN-γ expression and CYT scores, both indicating that cytotoxic function was efficiently elicited in those patients (Figure 1B). In our study, we followed inclusion criteria from several key clinical trials, such as CRYSTAL, FIRE-3, and PRIME (17, 27) to differentiate between the right (cecum to transverse colon) and left (splenic flexure to rectum) CRC with the splenic flexure; we treated rectum cancer as left-side CRC. Furthermore, we analyzed our data using only the non-rectal tissue and found that the immune landscape of the rectum and left-colon cancer microenvironments were not significantly different (Figure S1 in Supplementary Material). For this reason, rectum and left-colon cancer were considered as left-side CRC in our subsequent analysis.

The spectrum of somatic mutations in patients with the left- and right-side CRCs is known to be different (28). These preferences led us to test whether the infiltration pattern of immune cells is associated distinct mutations. The association between immune cell infiltration and the mutation status of *EGFR, BRAF, KRAS*, and *TP53* was assessed. More infiltration was observed in patients harboring mutations in *EGFR* (Figure S2A in Supplementary Material, H = 169; M = 235; L = 148 vs H = 26; M = 36; L = 24, *EGFR*-mutant vs non-mutant; *p* = 0.045) and *BRAF* (Figure S2B in Supplementary Material, H = 158; M = 214; L = 151 vs H = 47; M = 54; L = 14, *p* = 0.0008), supporting a genomic-immunophenotype correlation found in a recent analysis of melanoma (29). However, there was no relationship between *KRAS* or *TP53* mutation status and immune cell infiltration (Figures S3C,D in Supplementary Material). Consequently, the heterogeneous infiltration of immune cells and its correlation with genomic aberrations suggest that there are different immunophenotypes among patients with distinct primary tumor locations (PTLs).

### The Right-Side TME Is Associated With Increased Immune Cell Infiltration and Highly Cytotoxic Potential

**Figure 2:**
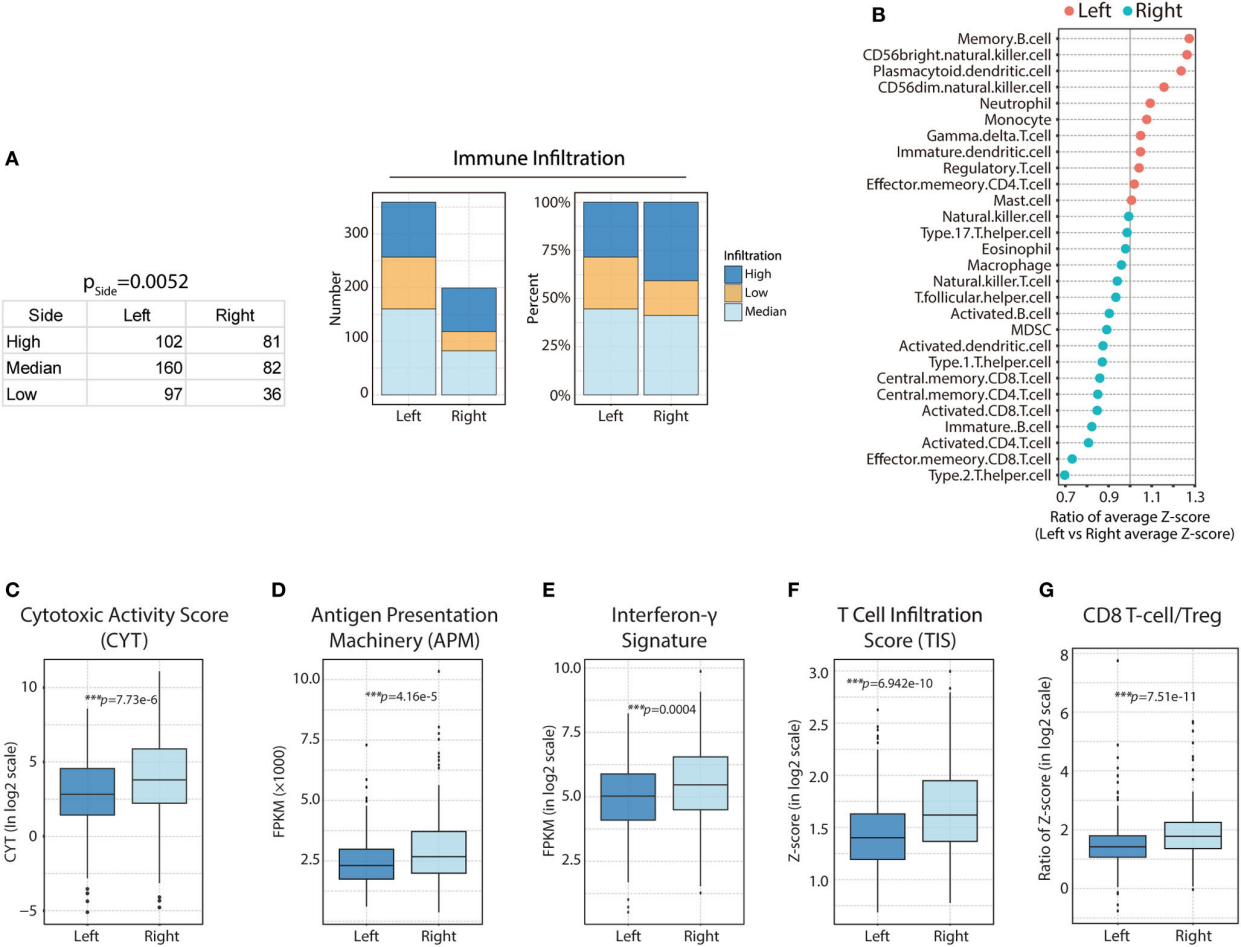
Heterogeneous immune cell infiltration in right-side and left-side colorectal cancer (CRC). **(A)** Immune infiltration in right-side and left-side CRC based on immune infiltration clustering scores [right side: high (H) = 81; median (M) = 82; low (L) = 36 vs left side: H = 102; M = 160; L = 97, *p* = 0.0052]; the absolute value and composition ratio of different immune infiltration statuses are show on the right. **(B)** Distribution of immune cell infiltration between different primary tumor locations. Higher than 1, more infiltration in left side; lower than 1, less infiltration in right side. **(C)** Comparison of relative cytotoxic activity scores (CYT) between right- and left-side CRC (*p* = 7.73e−6). **(D)** Relative antigen presentation machinery (APM) between right-side and left-side CRC (*p* = 4.16e−5). **(E)** Relative interferon-γ signature between right- and left-side CRC (*p* = 0.0004). **(F)** Relative T-cell infiltration score (TIS) between right- and left-side CRC (*p* = 6.942e−10). **(G)** Relative CD8 T-cell/Treg ratios between right- and left-side CRC (*p* = 7.51e−11). Two-tailed Student’s *t*-tests were used for all analyses. Error bars represent the mean ± SEM. *p < 0.05; **p < 0.01; ***p < 0.001; *p* ≥ 0.05, not significant.

First, we compared total immune cell infiltration among CRCs from different PTLs. Interestingly, we found that the degree of immune infiltration was higher in right-side colon cancer than in left-side CRC (Figure 2A; H = 81, M = 82, L = 36 in right side vs H = 102, M = 160, L = 97 in left side; *p* = 0.0052). For more sophisticated characterization, we compared each immune cell population based on their normalized ssGSEA score. Generally, subpopulations of T lymphocytes were found to be predominantly enriched in the TME from the right side, with the exception of effector memory CD4^+^ T-cells (Figure 2B). The association between the enrichment of T lymphocytes based on sidedness and higher immune cell infiltration was corroborated; specifically, enhanced cytotoxic function was identified in patients with higher immune cell infiltration (Figure 1B), suggesting highly cytotoxic potential in right-side tumors. To compare cytotoxic function between PTLs, the associated signatures were identified for each patient. Consistent with the higher infiltration of immune cells, right-side colon cancers were associated with higher levels of immune activation; specifically, cytotoxic activity score (CYT, *p* = 7.73e−6) (Figure 2C), antigen presentation machinery (APM, *p* = 4.16e−5) (Figure 2D), interferon-γ signature (*p* = 0.0004) (Figure 2E), TIS (*p* = 6.942e−10) (Figure 2F), and CD8^+^ T-cell/Treg ratio (*p* = 7.51e−11) were higher (Figure 2G). These data illustrate that compared with left-side tumors, right-side tumors have a distinct immune phenotype, characterized by more immune infiltration and higher levels of immune activation.

### Enhanced CD56^bright^ NK Cell Response in Left-Side CRC

Natural killer cells are effector lymphocytes of the innate immune system that control several types of tumors by limiting their growth and dissemination (30). In murine models, it has been shown that NK cells can control both local tumor growth and metastasis due to their ability to exert direct-cellular cytotoxicity, without prior sensitization, and to secrete immunostimulatory cytokines like IFN-γ, which helps to shape the immune TME by activating effectors of adaptive immunity (31). Nevertheless, NK cells display impaired functionality and capacity to infiltrate tumors in cancer patients. This suggests that NK cells are feasible targets for immunotherapeutic approaches such as antibody-based strategies (32).

**Figure 3:**
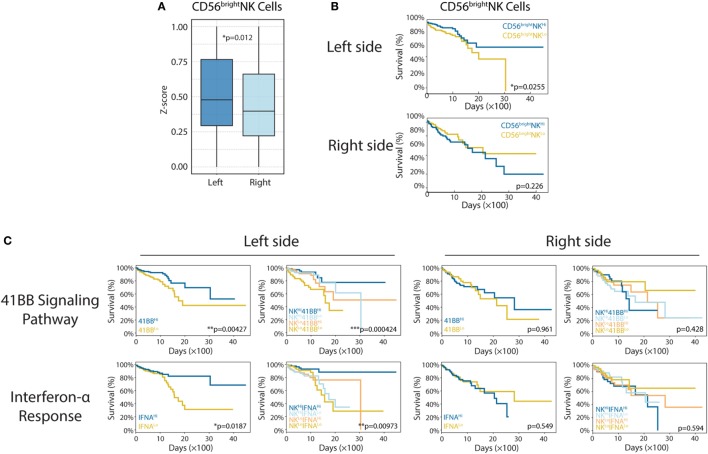
Enhanced CD56^bright^ natural killer (NK) cell response in left-side colorectal cancer (CRC). **(A)** CD56^bright^ NK cells in right- and left-side CRC (left vs right, *p* = 0.012). **(B)** CD56^bright^ NK cell infiltration was shown to correlate with patient survival [left: *n*(CD56^bright^ NK^Hi^) = 179, *n*(CD56^bright^ NK^Lo^) = 180, *p* = 0.0225; right: *n*(CD56^bright^ NK^Hi^) = 99, *n*(CD56^bright^ NK^Lo^) = 100, *p* = 0.226]. **(C)** The 4-1BB signaling pathway and interferon-α response have different roles in right- and left-side CRC. In the left side [4-1BB alone: *n*(4-1BB^Hi^) = 179, *n*(4-1BB^Lo^) = 180, *p* = 0.00427; IFNA alone: *n*(IFNA^Hi^) = 179, *n*(IFNA^Lo^) = 180, *p* = 0.0187; 4-1BB combined with CD56^bright^ NK cells: *n*(NK^Hi^4-1BB^Hi^) = 89, *n*(NK^Hi^4-1BB^Lo^) = 90, *n*(NK^Lo^4-1BB^Hi^) = 90, *n*(NK^Lo^4-1BB^Lo^) = 90, *p* = 0.000424; IFNA combined with CD56^bright^ NK cells: *n*(NK^Hi^ IFNA^Hi^) = 89, *n*(NK^Hi^ IFNA^Lo^) = 90, *n*(NK^Lo^ IFNA^Hi^) = 90, *n*(NK^Lo^ IFNA^Lo^) = 90, *p* = 0.00973], in the right side [4-1BB alone: *n*(4-1BB^Hi^) = 99, *n*(4-1BB^Lo^) = 100, *p* = 0.961; IFNA alone: *n*(IFNA^Hi^) = 99, *n*(IFNA^Lo^) = 100, *p* = 0.549; 4-1BB combined with CD56^bright^ NK cells: *n*(NK^Hi^4-1BB^Hi^) = 49, *n*(NK^Hi^4-1BB^Lo^) = 50, *n*(NK^Lo^4-1BB^Hi^) = 50, *n*(NK^Lo^4-1BB^Lo^) = 50, *p* = 0.428; IFNA combined with CD56^bright^ NK cells: *n*(NK^Hi^ IFNA^Hi^) = 49, *n*(NK^Hi^ IFNA^Lo^) = 50, *n*(NK^Lo^ IFNA^Hi^) = 50, *n*(NK^Lo^ IFNA^Lo^) = 50, *p* = 0.594]. Two-tailed Student’s *t*-tests and Kaplan–Meier survival log-rank test were used for those analyses. Error bars represent the mean ± SEM. *p < 0.05; **p < 0.01; ***p < 0.001; *p* ≥ 0.05, not significant.

In this study, we determined that left-side CRC was associated with higher levels of CD56^bright^ NK cell infiltration, compared with that in right-side colon cancer (left vs right, *p* = 0.012; Figure 3A). More strikingly, CD56^bright^ NK cell infiltration correlated strongly with patient survival in left-side CRC (left *p* = 0.0225; right *p* = 0.226; Figure 3B). It has been confirmed that CRC prognosis varies significantly based on PTL; patients with left-side tumors experience higher cure rates and superior OS (33). There are many molecular factors believed to contribute to improved survival in left-side CRC, many of which can be attributed to differences in genetic alterations. Our analysis revealed that major differences in immune infiltration, especially CD56^bright^ NK cells in left-side CRC, might also mediate some of these survival differences. Based on the aforementioned results, we hypothesized that enhanced CD56^bright^ NK cell activation and function in left-side CRC might also be beneficial for prolonged patient survival. Therefore, we performed additional analysis to examine whether the 4-1BB and interferon-α signaling pathways, which are known to reinforce the function of NK cells (34, 35), could also prolong OS for left-side CRCs. As expected, we observed significantly increased survival in patients with more active 4-1BB and interferon-α signaling pathways in left-side CRCs (Figure 3C; 4-1BB: *p* = 0.00427, IFNA: *p* = 0.0187 for left side and 4-1BB: *p* = 0.961, IFNA: *p* = 0.549 for right side). Furthermore, to test if higher levels of CD56^bright^ NKs and more active 4-1BB/interferon-α signaling could classify populations based on the best tumor control and extended survival, we performed survival analysis. We identified the most favorable OS in the NK^Hi^41BB^Hi^ and NK^Hi^IFNA^Hi^ cohort, further implying a critical role of NK subsets in antitumor immunity in left-side CRC (Figure 3C; CD56^bright^ NK cells combined with 4-1BB pathway, *p* = 0.000424, CD56^bright^ NK cells combined with IFNA pathway, *p* = 0.00973 for left side and CD56^bright^ NK cells combined with 4-1BB pathway *p* = 0.428, CD56^bright^ NK cells combined with IFNA pathway *p* = 0.594).

### Vascular Endothelial Growth Factor A (VEGF-A) Is Negatively Related to Cytotoxic Signatures and Predicts OS With CD8 Infiltration in Right-Side CRC

**Figure 4:**
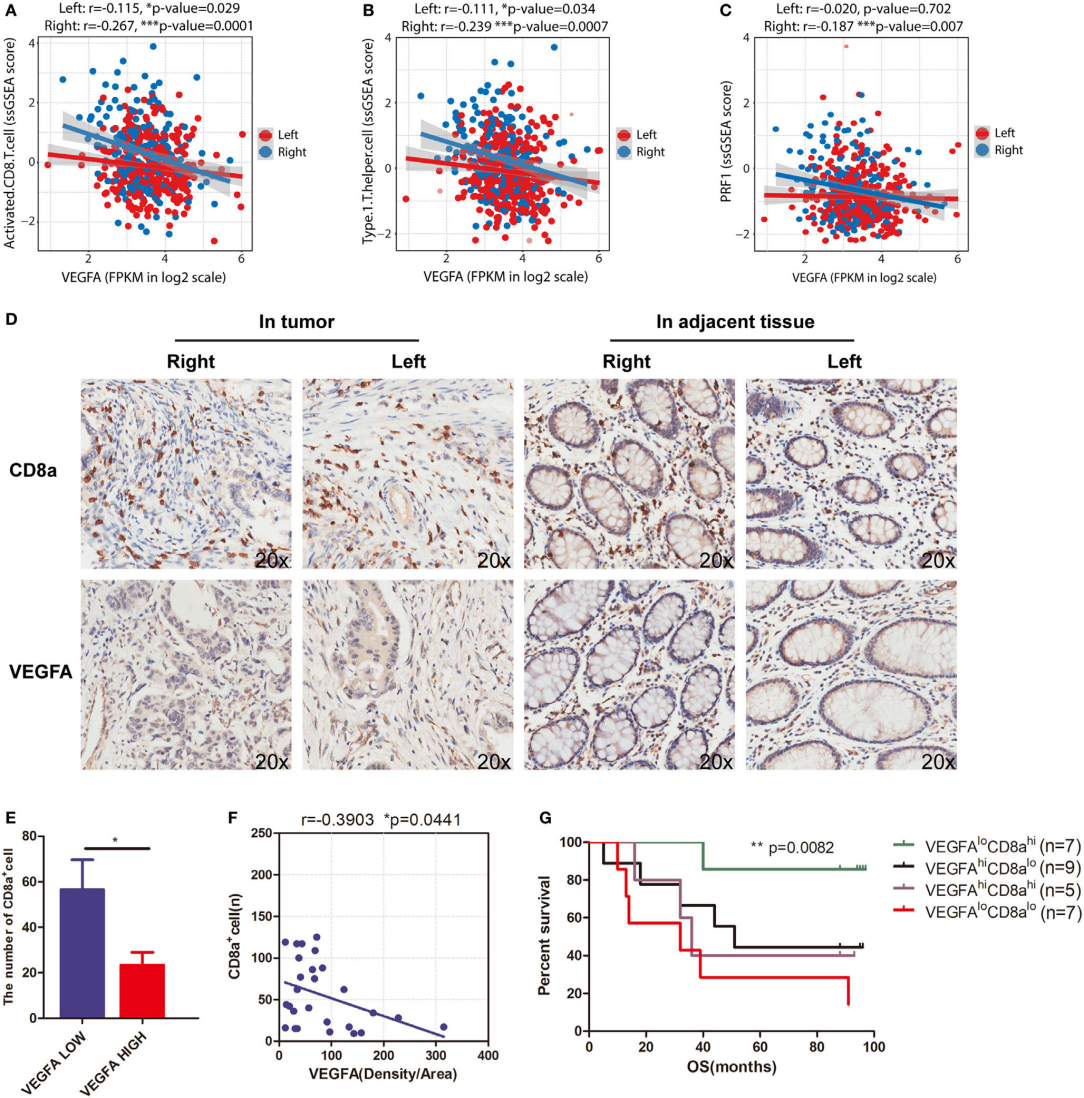
VEGFA hinders T-cell infiltration in right-side colorectal cancer (CRC). Correlations between **(A)** VEGFA and T-cell infiltration (right side: *r* = −0.267, *p* = 0.0001; left side: *r* = −0.115, *p* = 0.029), **(B)** VEGFA and Th1-cells (right side: *r* = −0.239, *p* = 0.0007; left side: *r* = −0.111, *p* = 0.034), and **(C)** VEGFA and perforin (PRF1) expression (right side: *r* = −0.187, *p* = 0.007; left side: *r* = −0.02, *p* = 0.702). **(D)** Infiltration of CD8 cells and expression of VEGFA detected in CRC tissue microarrays (20×). **(E)** The number of CD8 alpha-positive cells in the VEGFA low and high group in right-side tumors. The group was divided into two subgroups based on the median value (*p = 0.0386). **(F)** Correlation between VEGFA and CD8 alpha-positive cells in right-side tumors (*r* value represents Pearson correlation coefficient). Two-tailed *p*-values are presented for significance (<0.05; *r* = −0.3903, *p* = 0.0441; *n* = 28). **(G)** Kaplan–Meier curves for cancer-specific survival based on CRC immune infiltration classes. The CD8^hi^VEGFA^lo^ class had the best survival, whereas other classes were associated with poor outcome (log-rank test, *p* = 0.0082). Two-tailed Student’s *t*-tests and Kaplan–Meier survival log-rank test were used for those analyses. Error bars represent the mean ± SEM. *p < 0.05; **p < 0.01; ***p < 0.001; *p* ≥ 0.05, not significant.

The T-cell immune response is the central event in antitumor immunity. Effective immunotherapy requires that neoantigen-specific T-cells be generated and enter the tumor niche. Pathologic angiogenesis, which is mainly mediated by VEGF-A, prominently hinders T-cell infiltration (36, 37). In this study, we investigated sidedness differences for the correlation between VEGF-A expression and cytotoxicity signatures. For right-side tumors, we found that elevated VEGF-A expression correlated not only with the decreased level of activated CD8^+^ T-cells (Figure 4A, *r* = −0.267, *p* = 0.0001) and decreased Th1 cell infiltration (Figure 4B, *r* = −0.239, *p* = 0.0007) but also with the downregulation of perforin—a predominant molecular effector of cytotoxic activity (Figure 4C, *r* = −0.187, *p* = 0.007). At the same time, when this analysis was applied to the left-side type of the disease, much weaker correlations were identified, emphasizing the distinct contribution of angiogenesis to impaired infiltration and cytotoxicity (Figures 4A–C, red dots and lines). Although the three *p* values are less than 0.01 in right-side tumors, the correlation coefficients displayed are slightly. We therefore suggest that VEGFA negatively and significantly correlates with cytotoxic signatures in the right-side tumors when compared with the left-side.

To further validate the findings from TCGA data that VEGFA negatively and significantly correlates with activated CD8^+^ T-cell infiltration, we applied immunohistochemical staining of a TMA to analyze a CRC cohort (Figure 4D). In the right-side tumor, when patients were grouped based on VEGFA expression, we found a twofold decrease in CD8A expression, and observed a negative correlation (*r* = −0.3903, *p = 0.0441) between VEGFA and CD8A expression in right-side colon cancer (Figures 4E,F). This suggests an interaction between VEGFA and CD8^+^ T-cells in patients with right-side CRC. This VEGFA-CD8A correlation further leads us to hypothesize a CD8A^Hi^VEGFA^Lo^ cohort with good prognosis among right-side CRCs. As expected, prolonged survival in CD8A^Hi^VEGFA^Lo^ patients was found (**p = 0.0082, Figure 4G). Taken together, we identified that VEGFA expression is negatively related to CD8A expression and favorable outcome for CD8A^Hi^VEGFA^Lo^ patients, implying that the antitumor immune microenvironment, and especially the infiltration of CD8^+^ T-cells, could feasibly be restored by anti-angiogenic treatment.

## Discussion

To the best of our knowledge, this represent the first depiction of the immune landscape of CRCs identified using a large cohort. More importantly, we found that left-side CRC and right-side colon cancer have distinct immune landscapes, and that this immune landscape might, to some extent, explain the primary CRC location-specific differences in prognoses and responses to anti-VEGF and anti-EGFR agents.

In our analysis, we found that there was higher degree of infiltration of the antitumor CD56^bright^ NK cell subpopulation in left-side CRC. Moreover, CD56^bright^ NK cell infiltration significantly correlated with patient survival. These findings might explain the clinical phenomenon that patients with RAS wild-type, left-sided primary tumors show better response to EGFR inhibitors (cetuximab) than right-sided tumors (FIRE-3 trial) (27). The Fc fragment of cetuximab can bind the Fc receptor FcγRIII (CD16) on NK cells; this initiates a sequence of cellular events culminating in the release of cytotoxic granzyme-containing granules and INF-γ secretion, which subsequently kills the tumor cells (38). By contrast, we found that activation of the CD137 (4-1BB) receptor signaling pathway was positively associated with patient survival in left-side CRC. This result further supports the recent findings that targeting CD137 enhances cetuximab efficacy (39). More recently, it was found that the PD-1 receptor can also be detected on activated NK cells (40, 41), suggesting that blocking this protein could facilitate CD56^bright^ NK cell cytolytic activity. Therefore, our present findings suggest a new strategy, namely, the combination of anti-PD-L1 antibody with cetuximab, for the treatment of left-side, RAS wild-type CRC. Meanwhile, in the part of survival analysis, we only took into account the effect of NK on OS. This may potentially circumvent the need to target complex and evolving somatic mutational spectrum, such as EGFR, BRAF, *p*53 mutation status, and mismatch-repair deficiency.

To the best of our knowledge, the T-cell immune response is the central event in antitumor immunity (42, 43). Fortunately, we observed enhanced CD8^+^ T-cell infiltration, cytotoxic activity, and interferon-γ signature and APM in right-side CRC compared with those in left-side CRC. In some cases, right-side CRC and left-side CRC show various biological and clinical differences including embryonic origin, microbiota burden, vascular supply, and main physiologic function (2), which may affect somatic mutations and immune phenotypes between the two different disease locations. These immune phenotypes are consistent with the established knowledge that more mutations are typically found in right-side disease (44–46). However, whether there exists a different expression profile of MHC class I molecules among patients with distinct PTLs need to be further explored. Moreover, this beneficial CD8^+^ T-cell-mediated antitumor response might be blocked by high concentrations of VEGF-A, which not only mediates immune tolerance but also restricts T-cell infiltration into the tumor (47–49). In addition, an analysis of the genomic and transcriptomic features of metastatic melanoma suggests that angiogenesis is enriched in non-response to anti-PD-1 therapy (50). Taken together, these data reveal the paradoxical observation that despite more efficient CD8^+^ T-cell infiltration, cytotoxic activity, and interferon-γ signature and APM, right-side CRC have worse prognosis.

Our present results clearly showed that CD8^+^ T-cell infiltration is negatively associated with VEGF-A expression in right-side CRC (Figure 4A, *r* = −0.267, *p* = 0.0001). In the analysis of CRC cohort, we also identified that VEGFA is negatively related to CD8A and favorable outcome for CD8A^Hi^VEGFA^Lo^ patients. These findings might explain the clinical phenomenon that patients with these primary tumors respond to VEGF inhibitors (bevacizumab) better than those with left-side tumors (PEAK and CALGB/SWOG 80405 trials) (51). At the same time, tumor display generally higher levels of T cell infiltration but also higher levels of immunoregulatory influence as represented by their PD-1/PD-L1 (52, 53). In view of these results, we suggest the combination of anti-PD-1 or anti-PD-L1 antibodies and VEGFA inhibition should be of particular interest for the treatment of right-side colon cancer. Coincidentally, the combination of ANGPT2 and VEGFA inhibition and PD-1 blockade was shown to improve tumor control, supporting the rationale for co-targeting angiogenesis and immune checkpoints for colon cancer therapy (54). Ongoing clinical trials of ANGPT2 and VEGFA inhibitors in combination with the anti-PD-L1 monoclonal antibody atezolizumab (NCT01688206^3^) will provide important information on the benefits of such combination.

**Figure 5:**
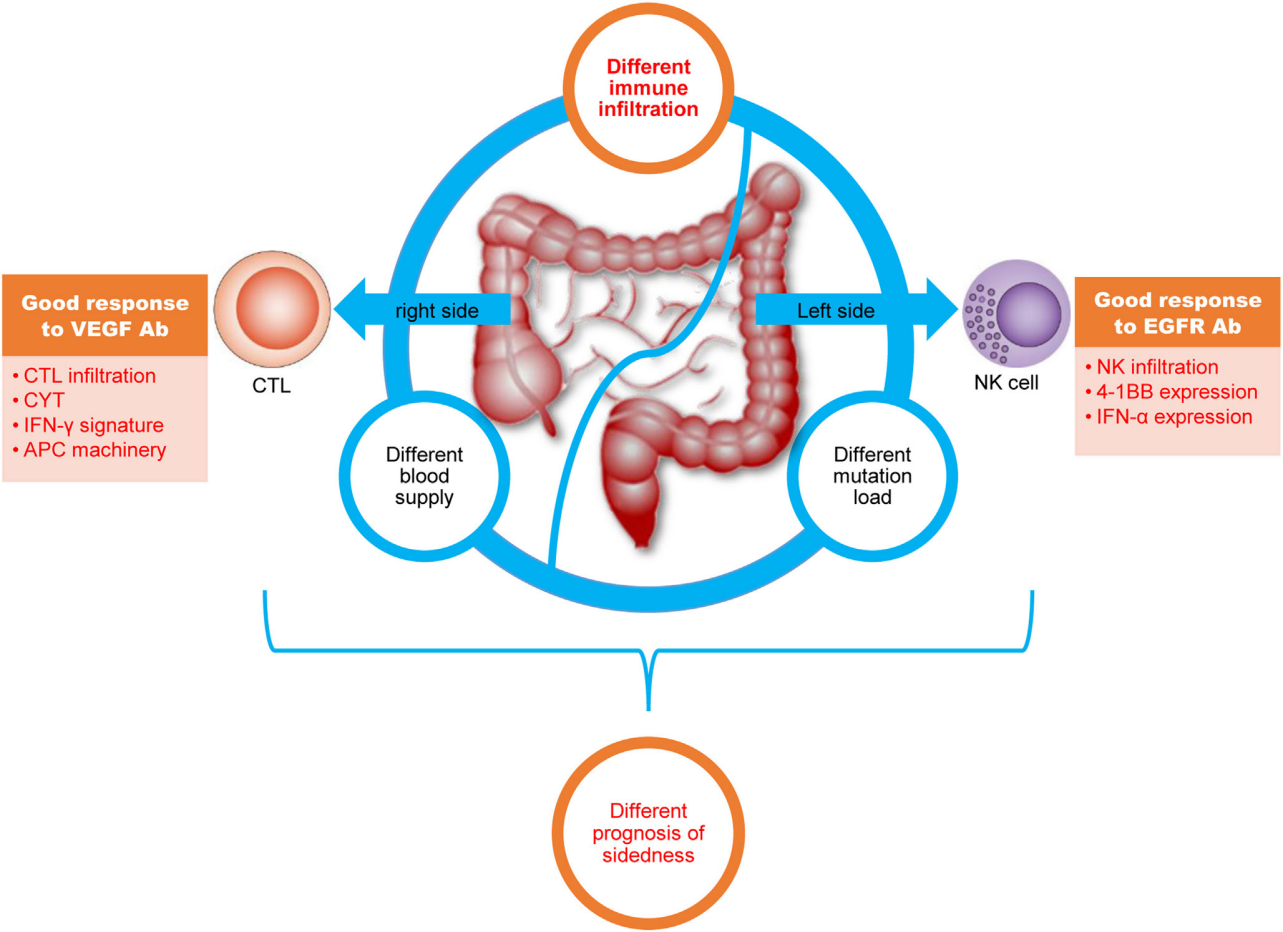
The colorectal cancer (CRC) immune landscape and implications for immunotherapy. Right-side colon (cecum to transverse colon) cancer and left colorectal (splenic flexure to rectum) cancer have different mutation loads, immune infiltration, and blood supplies; furthermore, different immune cell infiltration profiles, signaling pathways, and effector molecule in right-side colon cancer and left-side CRC might lead to different treatment responses. All of these differences might lead to different prognoses with immunotherapy.

Overall, our depiction of the immune landscape of CRC is highly important to clinically relevant processes (Figure 5). First, in addition to differences in mutation load and blood supply, we present disparity in immune cell infiltration and immune phenotypes between right- and left-side CRC. These newly identified differences might, to some extent, contribute to the different prognoses between the two different disease locations. Second, predominant CD8^+^ T-cell infiltration in right-side colon cancer and enhanced NK infiltration in left-side CRC explains the elusive clinical phenomenon that patients with right-side disease have good response to anti-VEGFA antibodies, whereas those with left-side CRC have good response to anti-EGFR antibodies. Third, regarding anti-PD-1 or anti-PD-L1 antibody immunotherapy, our results suggest for CRC with wild-type RAS, PD-1/PD-L1 blockage should be combined with anti-VEGF antibodies for right-side disease and with anti-EGFR antibodies for left-side disease.

## Author Contributions

BZ and QJ conceived and designed the experiments. LZ, YZ, YD, J-NC, ZG, and YF wrote scripts for data analysis. QJ and CS analyzed and interpreted data. QJ and BZ wrote the manuscript.

## Conflict of Interest Statement

The authors declare that the research was conducted in the absence of any commercial or financial relationships that could be construed as a potential conflict of interest.

## Footnotes

^1^http://gdac.broadinstitute.org/ (Accessed: April 18, 2017).

^2^https://gdc-portal.nci.nih.gov/ (Accessed: April 18, 2017).

^3^www.clinicaltrials.gov (Accessed: April 18, 2018).
