# Supplementary material for: Immune Landscape of Colorectal Cancer Tumor Microenvironment from Different Primary Tumor Location
Source: Front Immunol. 2018 Jul 10;9:1578. doi: 10.3389/fimmu.2018.01578 (PMC6048410; doi:10.3389/fimmu.2018.01578)
Supplement: Supplementary file 1 [file Presentation_1.pdf]

Supplementary Material

Immune Landscape of CRC Tumor Microenvironment from Different Primary Tumor Location

Longhui Zhang[1], Yuetao Zhao[1], Ying Dai[1], Jia-Nan Cheng[1], Zhihua Gong[1], Yi Feng[1], Chengdu Sun[1], Qingzhu Jia[1*], Bo Zhu[1, 2*]

Figure S1. Cytotoxic function for high, median, and low immune infiltration among non-rectal tumors clustered on the basis of immune cell infiltration.

Figure S2. The relation between immune infiltration and *EGFR, BRAF, KRAS* and *TP53* mutation status.

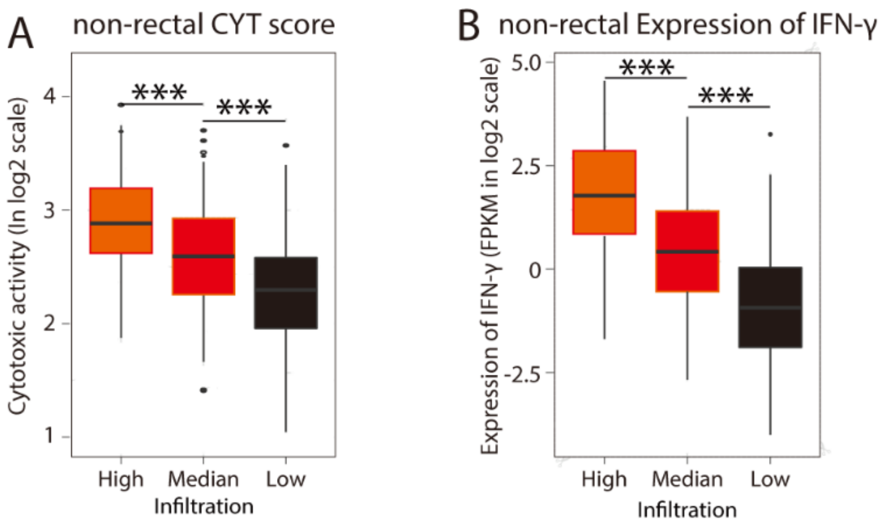

Figure S1. Cytotoxic function for high, median, and low immune infiltration among non-rectal tumors clustered on the basis of immune cell infiltration.
(A) Relative cytolytic score (CYT), and **(B)**expression of IFN-γ for high, median, and low immune infiltration among non-rectal tumors clustered by overall immune cell infiltration. Two-tailed Student's t-tests were used for all analyses. The error bars represent the means ± SEM. $*p < 0.05$; $**p < 0.01$; $***p < 0.001$; $p \geqslant 0.05$, not significant.

A

p_{EGFR}=0.045

EGFR	Mutation	Wildtype
High	26	169
Median	36	235
Low	24	148

B

p_{BRAF}=0.0008

BRAF	Mutation	Wildtype
High	47	158
Median	54	214
Low	14	151

C

p_{KRAS}=0.499

KRAS	Mutation	Wildtype
High	75	139
Median	93	168
Low	66	97

D

p_{TP53}=0.5660

TP53	Mutation	Wildtype
High	120	130
Median	137	124
Low	89	83

Figure S2. The relation between immune infiltration and *EGFR, BRAF, KRAS* and *TP53* mutation status.

(A) The degree of immune infiltration in *EGFR* wild type is higher than *EGFR* mutation (H=169; M=235; L=148 vs H=26; M=36; L=24, *p*=0.045). **(B)**The same to *BRAF* genotype, wild type is higher than mutation (H=158; M=214; L=151 vs H=47; M=54; L=14, *p*=0.0008) . **(C, D)**The relationship between *KRAS* and *TP53* mutation status and immune cell infiltration are negative.
